# Qualitative beta-2-adrenoceptor signaling in the regulation of human airway epithelia mucin and cytokine production

**DOI:** 10.1186/s12931-026-03693-4

**Published:** 2026-05-01

**Authors:** Fred Graumuller, Diana Cervantes, Tung O. Chan, Niccolette Schaunaman, Dominic R. Villalba, Burton F. Dickey, Julia K. L. Walker, Hong Wei Chu, Raymond B. Penn

**Affiliations:** 1 Department of Medicine, Pulmonary and Critical Care Medicine, Center for Translational Medicine, Jane and Leonard Korman Respiratory Institute, Thomas Jefferson University, Philadelphia, PA 19107 USA; 2 Department of Medicine, National Jewish Health, Denver, CO 80206 USA; 3 Department of Pulmonary Medicine, University of Texas MD Anderson Cancer Center, Houston, TX USA; 4 Department of Medicine and School of Nursing, Duke University Medical Center, Durham, NC USA

**Keywords:** Asthma, Inflammation, Protein kinase A, Beta-arrestin, G protein, Biased signaling

## Abstract

**Background:**

Numerous in vivo studies have demonstrated beta-2-adrenoceptor (β_2_AR) -agonism as permissive in the development of allergic lung inflammation, and have implicated the arrestin-dependent signaling arm of the β_2_AR in mediating this effect. However, the specific cell type(s) mediating β_2_AR regulation of allergic lung inflammation remain unestablished.

**Methods:**

To explore the potential contribution of airway epithelia in this phenomenon, we compared the ability of ractopamine (RP), recently identified as a Gs-biased beta-agonist, to that of the unbiased/balanced beta-agonist albuterol (ALB), on IL-13-stimulated mucin and cytokine production in human airway epithelia cultures in air–liquid interface (HAE). Results: ALB, which activates both the β_2_AR-arrestin and -Gs signaling pathways significantly augmented IL-13-induced mucin production in HAE. RP, which preferentially signals via Gs/PKA, did not. Although IL-13 stimulated production of numerous cytokines, including IL-1α, IL-1RA, MDC, TGF-α, and GROα, ALB-mediated augmentation of these cytokines was highly variable and not statistically significant. Similarly, RP did not augment the induction of cytokines stimulated by IL-13. Moreover, in contrast to previous studies that reported a requirement of concomitant β_2_AR agonism for IL-13 to stimulate cytokine production, such a requirement was observed only in minority of the (12) cultures examined.

**Conclusions:**

These data implicate arrestin-dependent β_2_AR signaling augmenting airway epithelial mucin production as a contributor to the previously-demonstrated pro-inflammatory effects of β_2_AR agonism in vivo. Moreover, they suggest that beta-agonist effects on the cytokine profile in the allergen-inflamed lung may be influenced by specific asthmatic endotypes and involve cooperativity among multiple cell types.

**Supplementary Information:**

The online version contains supplementary material available at 10.1186/s12931-026-03693-4.

## Introduction

Agonists of the beta-2-adrenoceptor (β_2_AR), *beta-agonists*, are fundamental to asthma treatment, as they are effective as both prophylaxis and rescue medication for many asthmatics. However, 30 + years of basic, translational, clinical, and epidemiological research reveal they provide suboptimal control for up to 50% of asthmatics [1, 2]. Moreover, lingering safety concerns regarding long-acting β-agonists (LABAs) necessitate the preferential or concomitant use of corticosteroids. Over the last two decades numerous studies, most employing genetic and molecular approaches in murine models of allergic lung inflammation, have convincingly demonstrated that both endogenous (epinephrine) and exogenous (e.g., albuterol, formoterol) beta-agonists promote the asthma phenotype [3–9], and that mice lacking the β_2_AR fail to develop an asthma phenotype in allergen or IL-13 models of allergic lung inflammation [3, 5, 6], and that the β_2_AR regulatory and signaling protein arrestin is critical to the limited efficacy and safety issues associated with β-agonist use in asthma [10, 11]. However, although such work to date constitutes compelling proof of principle, like much of asthma research, it has yet to establish a corrective pharmacological approach sufficiently feasible to advance to the clinic.

Recent advancements in understanding qualitative/biased G protein-coupled receptor signaling and the emergence of *biased ligand pharmacology* offer hope that new β_2_AR ligands, be they orthosteric agonists or allosteric modulators, will be more efficacious and safe than currently approved beta-agonists [12, 13]. In recent years a handful of β_2_AR ligands that promote Gs-biased signaling have been identified. These include a negative allosteric modulator (NAM) of β_2_AR arrestin signaling [14], and positive allosteric modulators (PAM) of β_2_AR Gs signaling [15]. In addition, using a comprehensive library screen De Pascali et al. identified orthosteric β_2_AR agonists capable of stimulating β_2_AR Gs signaling without recruiting arrestin to the β_2_AR [16]. Although the identified Gs-biased β_2_AR orthosteric agonists lack β_2_AR specificity and thus are not suitable asthma drugs, they nevertheless represent useful tools to test effects of β_2_AR bias in model systems not amenable to molecular or genetic manipulation, while also enabling preclinical analysis of drugs that may be refined by medicinal chemistry.

Airway epithelium is a key immunomodulatory cell in the lung, and Nguyen et al. previously determined that in β_2_AR-/- mice, transgenic expression of the β_2_AR only in airway epithelia was sufficient to rescue IL-13-induced airway hyperresponsiveness, inflammation, and mucin production [5]. To further explore the role of epithelia, and qualitative β_2_AR signaling, in a more relevant human model, we compared the effects of the Gs-biased beta-agonist ractopamine (RP) to that of the unbiased beta-agonist albuterol (ALB), on HAE stimulated chronically with IL-13 in the air–liquid interface (ALI) culture model.

## Methods

### Establishment, culture and treatment of HAE

Cultures were generated from harvested epithelia from bronchial epithelial brushes as per [17] from either non-asthmatic or asthmatic subjects. Asthma was diagnosed by NJH physicians based on the criteria described in the Global Initiative for Asthma Main Report [18]. All subjects underwent informed consent and procedures/studies were approved by the NJH IRB. Because HAE cultures were generated from banked and deidentified cells, the proposed work is judged to be Not Human Subjects Research by TJU, Duke University and NJH IRB.

**Fig. 1:**
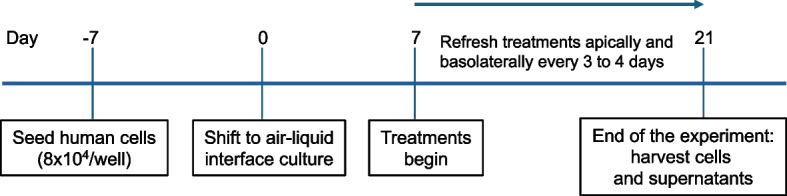
Experimental design of HAE culture, treatment, and terminal harvesting of basolateral supernatants or apical lavages. See *Methods* for details

The overall Experimental Design for HAE culture, treatment, and harvesting for analysis of mucin and cytokine production is schematically presented in Fig. 1. Briefly, cells were expanded by culturing on irradiated 3T3 fibroblast feeder cells in the presence of Rho kinase inhibitor Y-27632 (10 μM) as per [17]. Expanded HAE were trypsinized and seeded onto collagen-coated 12-well transwell plates with serum-free airway epithelial cell growth basal medium (PromoCell C-21260) supplemented with an equal volume of DMEM medium, bovine pituitary extract (0.004 ml/ml), insulin (5 µg/ml), hydrocortisone (0.5 µg/ml), epidermal growth factor (10 ng/ml), transferrin (10 µg/ml), retinoic acid (30 ng/ml), gentamicin (5 ug/ml), amphotericin B (0.12 µg/ml), bovine serum albumin (0.5 µg/ml), ethanolamine (50 mM), MgCl_2_ (3 mM), MgSO_4_ (4 mM) and CaCl_2_ (1 mM). Cells were grown for 7 days in submerged conditions in media containing Y-27632 and then shifted to ALI condition without the inhibitor in the media as per [17]. Media in wells were changed every 3–4 days. Starting at 7 days of the ALI culture, cells were treated with media containing vehicle or IL-13 (10 ng/ml) in the presence of vehicle, ALB (1 μM) or RP (1 μM) in both basolateral and apical compartments. After 14 days of treatment, basolateral supernatants were collected for cytokine analysis as detailed below. Supernatants for the apical compartment were generated by rinsing the apical surface with 100 µl of PBS for MUC5AC dot blot analysis as per below.

### Analysis of beta-agonist-stimulated Gs/PKA signaling

HAE cultures, generated and subjected to ALI culture 21 days without additional treatments, were acutely stimulated for 30 min with 10 ng/ml IL-13 ± vehicle, ALB (1 µM) or RP (1 µM), then cells were washed 3X with cold PBS and lysates generated by addition of a detergent lysis buffer optimized for analysis using the ProteinSimple Wes blotting system (see below). The lysis buffer consisted of equal volumes of an NP40-based buffer (1% NP40, 2% glycerol, 10 mM NaF, 137 mM NaCl, 20 mM Tris–HCl pH 8.0) and a RIPA lysis buffer (1% NP40, 0.5% sodium deoxycholate, 0.1% SDS, 150 mM NaCl, 50 mM Tris–HCl pH 8.0) with protease and phosphatase inhibitor cocktail (BioTools, Jupiter, FL, USA). For analysis of protein/phospho-protein expression, the ProteinSimple Wes (ProteinSimple, San Jose, CA, USA) system was used as described previously [19]. Wes is a western blotting alternative based on capillary electrophoresis immunoassay principle. The processes of protein fractionation, immobilization, and immunodetection are fully automated. The procedure was performed according to manufacturer’s protocol. Briefly, cell lysates were mixed 4:1 (vol/vol) with a master mix (final concentration of 40 mM dithiothreitol) and then heated at 95 °C for 5 min. The samples, antibody diluent, primary antibodies, secondary antibodies, chemiluminescent substrate, and wash buffer were added to designated wells in the manufacturer-provided microplate. After plate loading, the separation electrophoresis and immunodetection were performed using instrument default settings. Data were analyzed using the manufacturer-provided Compass software. Peak area of the target-protein chemiluminescence signal was calculated and normalized to the β-actin peak-area signal in the same sample. MWs of the proteins were calculated from the 3 MW markers added to each sample (as a component of the master mix), which were compared with a standard curve; 6 biotinylated proteins, ranging from 12 to 230 kDa, which were separated in a separate capillary and detected using streptavidin conjugated–horseradish peroxidase. Representative data are presented as an electropherogram of the chemiluminescence signal in the capillary.

### Analysis of beta-agonist-stimulated cAMP accumulation in BEAS-2B epithelial cells

To further characterize the pharmacological properties of ALB and RP, ALB- and RP- stimulated cAMP accumulation was assessed on the human epithelial cell line BEAS-2B using a protocol similar to detailed in Penn et al. [20] with experimental details provided in Supplemental Materials.

### Quantification of mucin, cytokine production

Supernatant levels of mucin were quantified by dot blot analysis as described previously [21]. Briefly, 25 µl of apical supernatants collected at day 21 of ALI culture was boiled at 90 °C for 10 min, and then transferred onto a 0.45um nitrocellulose membranes, which were probed with a mouse anti-human MUC5AC antibody (Novus biologicals, Centeniall, CO). Thereafter, blots were incubated with horseradish peroxidase-linked secondary mouse antibody and developed with enhanced chemiluminescence western blotting substrate. Densitometry was performed using the NIH ImageJ software.

The initial screening of cytokine expression in IL-13-stimulated basolateral supernatants from cultures derived from 5 different donors was assessed in a Plex Discovery Assay® Array assays by Eve Technologies (Calgary, Canada) covering 71 different cytokines. The Human Cytokine 71- Plex contained the following cytokines: sCD40L | EGF | Eotaxin | FGF-2 | Flt-3 ligand | Fractalkine | G-CSF | GM-CSF | GROα | IFNα2 | IFNγ | IL-1α | IL-1β | IL-1RA | IL-2 | IL-3 | IL-4 | IL-5 | IL-6 | IL-7 | IL-8 | IL-9 | IL-10 | IL-12p40 | IL-12p70 | IL-13 | IL-15 | IL-17A | IL-17E/IL-25 | IL-17F | IL-18 | IL-22 | IL-27 | IP-10 | MCP-1 | MCP-3 | M-CSF | MDC (CCL22) | MIG | MIP-1α | MIP-1β | PDGF-AA | PDGF-AB/BB | RANTES | TGFα | TNFα | TNFβ | VEGF-A | 6CKine | BCA-1 | CTCK | ENA-78 | Eotaxin-2 | Eotaxin-3 | I-309 | IL-16 | IL-20 | IL-21 | IL-23 | IL-28 | IL-33 | LIF | MCP-2 | MCP-4 | MIP-1δ | SCF | SDF-1α β | TARC | TPO | TRAIL | TSLP.

Subsequent analysis, using cultures derived from 12 different donors (7 asthmatic, 5 non-asthmatic) focused on 5 cytokines relevant to allergic lung inflammation that exhibited a > two-fold induction by IL-13 (IL-1α, IL-1RA, MDC, TGFα, IL-13 and GROα), for assessment of the regulatory effects of ALB and RP on IL-13-mediated induction using a custom 5 plex assay by Eve Technologies.

### Statistical analysis

Parametric data were analyzed using one-way ANOVA, followed by Tukey’s post-hoc test. Non-parametric data were analyzed using the Kruskal–Wallis test with Dunn’s post hoc analysis. Mann–Whitney test for two-group comparisons. A *p* value < 0.05 was considered statistically significant.

## Results

### ALB and RP stimulate comparable PKA activity in HAE

**Fig. 2:**
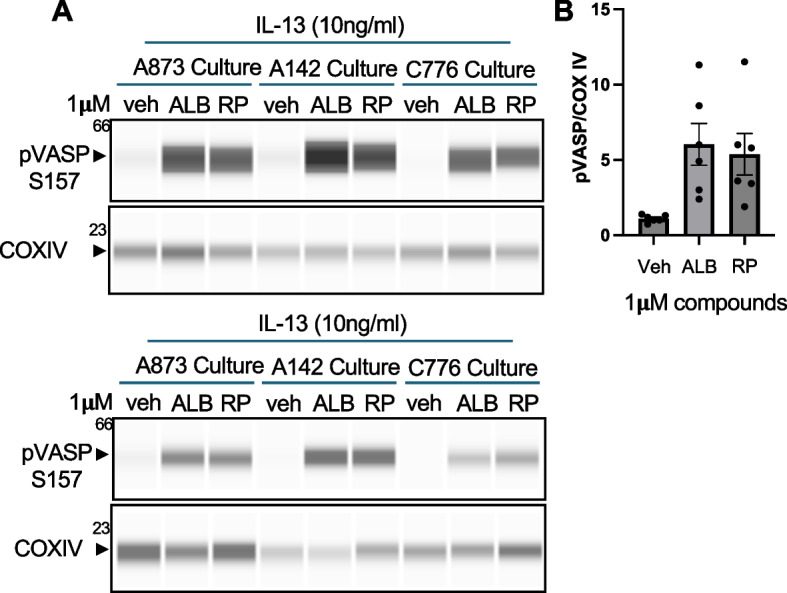
Albuterol—(ALB) and ractopamine (RP) –mediated induction of VASP phosphorylation. **A** HAE in ALI culture were stimulated with 10 ng/ml IL13 ± vehicle (Veh), 1 μM ALB, or 1 μM RP for 30 min. Cell lysates were generated and pVASP levels assessed using the ProteinSimple WES system with automated analysis (digital images and tracings shown in Supplemental Fig. 1). COXIV levels were assessed as a loading control. Images represent 2 different experiments from wells from 3 distinct cultures. **B** Graphic representation of mean ± SEM values; *n* = 6

De Pascali et al. previously identified RP as a Gs-biased beta-agonist based on its: 1) ability to stimulate Gs using the GloSensor cAMP reporter; and 2) inability to recruit arrestin to the β_2_AR in a standard BRET assay, in HEK293 cells with stable overexpression of the β_2_AR [16]. Although primary cultures of human airway epithelial cells expressing endogenous β_2_AR are not amenable to reporter/BRET analyses, we were able to establish that RP-stimulated Gs signaling to PKA is comparable to that of the β_2_AR agonist albuterol in HAE cultured under ALI with concomitant IL-13 treatment. Phosphorylation of the PKA substrate VASP has proven to be a highly sensitive indicator of PKA activity stimulated by the Gs/cAMP pathway [22, 23]. In HAE cultures derived from 3 different donors, stimulation with RP and ALB promoted similar levels of pVASP induction (Fig. 2, Supplemental Fig. 1). In addition, in BEAS-2B airway epithelial cell cultures, ALB and RP stimulated comparable levels of intracellular cAMP accumulation, in a dose-dependent manner (Supplemental Fig. 1).

### ALB, but not RP, increases IL-13-stimulated mucin in HAE

**Fig. 3:**
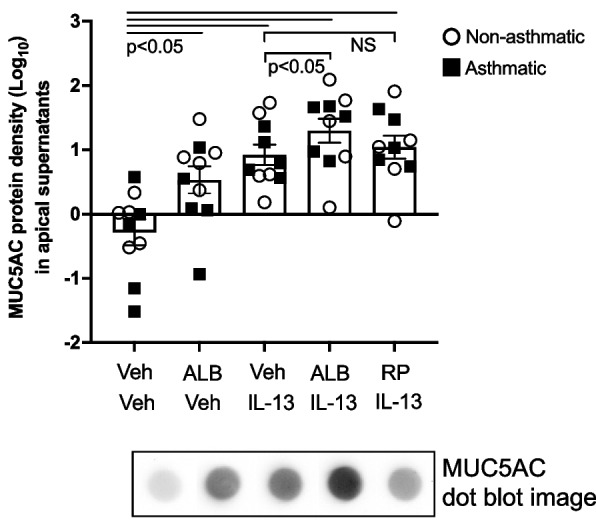
Albuterol (ALB) and ractopamine (RP) regulation of mucin levels in apical supernatants of ALI-cultured HAE from both non-asthmatic (open circles) and asthmatic (filled squares) subjects. ALB (1 μM) + IL-13, but not RP (1 μM) + IL-13, significantly increased secreted mucin levels as compared to control (- = Veh) and IL-13. ALB alone also significantly increased mucin secretion. Data were expressed as means from *n* = 5 healthy and *n* = 5 asthmatic subjects, and analyzed by one-way ANOVA, followed by Tukey’s post-hoc test. *, ALB vs. Veh, *p < 0.05, IL-13 vs. Veh, *p < 0.001; ALB + IL-13 vs. ALB, **p < 0.05, ALB + IL-13 vs. Veh, *p < 0.001

We next compared the ability of RP vs ALB in regulating IL-13 induction of mucin (MUC5AC). HAE cultures maintained in ALI were treated chronically with IL-13 plus either vehicle, ALB, or RP for 21 days, with media + agents refreshed every other day. MUC5AC levels were analyzed in apical side media at day 21 by dot blot analysis as per *Methods*. Consistent with prior findings [24, 25], IL-13 significantly induced mucin levels (Fig. 3). Moreover, co-treatment with ALB significantly augmented mucin levels stimulated by IL-13. However, co-treatment with the (Gs-biased) RP did not. Surprisingly, ALB treatment alone (in the absence of IL-13) significantly increased MUC5AC levels. No difference in the regulatory effect of IL-13 or beta-agonists on MUC5AC was observed between cultures derived from asthmatic (*n* = 5) or non-asthmatic donors (*n* = 5).

### IL-13 induces production of numerous cytokines in HAE; regulatory effects of beta-agonist are highly variable among cultures

**Fig. 4:**
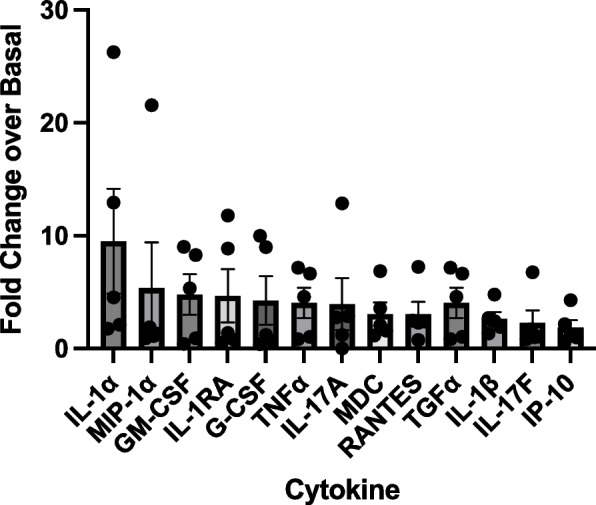
IL-13-induced cytokines in HAE cultures. Low passage bronchial brushing cells isolated from human subjects were expanded, grown and differentiated in air–liquid-interface transwell culture. Cells were then treated with IL-13 for 14 days. The culture media (*n* = 5) were collected and analyzed for a panel of 71 cytokines by antibody-based multiplexing laser beads. Mean ± SEM data are shown for those cytokines exhibiting a > two-fold change from basal

We next assessed the ability of IL-13 to induce cytokine production in HAE cultures, and compared the ability of RP vs ALB in regulating this effect. Initially, we screened 71 cytokines in 5 HAE cultures to identify cytokines that were induced by IL-13 (see *Methods*). We detected 13 cytokines with a > two-fold increase (Fig. 4) and noted that the cytokine induction by IL-13 varied widely among cultures.

**Fig. 5:**
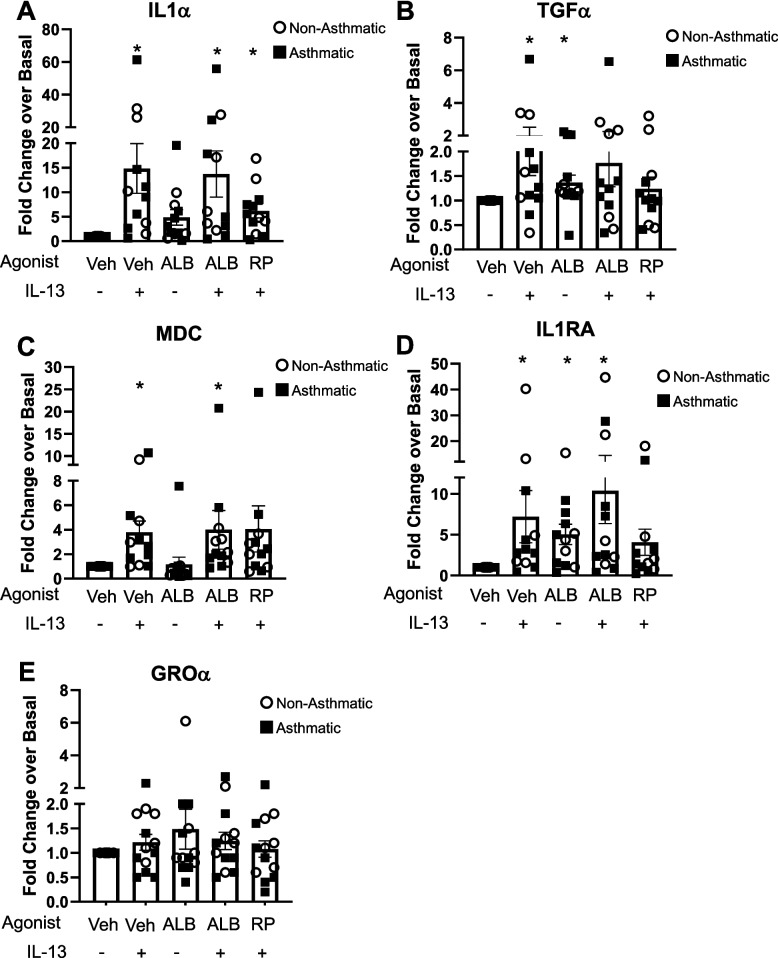
Albuterol (ALB) and ractopamine (RP) regulation of IL-13-induced cytokines. HAE cells in ALI culture were treated with IL-13 ± vehicle, 1 μM ALB, or 1 μM RP. The culture media were collected and analyzed for IL-1⍺, MDC, TGF⍺, IL-1RA and GRO⍺ expression by antibody-based multiplexing laser beads. Graph shows cytokine level normalized to basal (mean ± SEM values) from 12 different cultures. Individual data from experiments using HAE cultures derived from non-asthmatic (open circles) or asthmatic (filled squares) are presented for each condition. *p < 0.05, Veh vs. indicated stimuli

Using the same multiplexing technology, we next assessed the regulatory effect of ALB and RP on five cytokines induced by IL-13 in our preliminary screen and relevant to asthma pathology (IL-1α, IL-1RA, MDC, TGF-α, and GROα) in cultures derived from 12 different donors. In this analysis, IL-13 again significantly induced IL-1α, IL-1RA, MDC, and TGF-α, but not GROα (*p* < 0.05, Fig. 5), with data for each cytokine exhibiting high variability. Interestingly, ALB did not significantly increase the induction by IL-13 for any of the cytokines: (All mean ± SEM values: IL-1α: 15 ± 5, with ALB 14 ± 5, *p* > 0.99; IL-1RA: 7 ± 3, with ALB 10 ± 4, *p* > 0.99; MDC: 4 ± 1, with ALB 4 ± 2, *p* > 0.99; TGF-α: 2.0 ± 0.5, with ALB 1.8 ± 0.5, *p* > 0.99; GROα: 1.2 ± 0.2, with ALB 1.3 ± 0.2, *p* > 0.99). Similarly, RP did not significantly increase the induction by IL-13 for any of the cytokines: (IL-1α: 15 ± 5, with RP 6 ± 1, *p* = 0.47; IL-1RA: 7 ± 3, with RP 4 ± 2, *p* = 0.43; MDC: 4 ± 1, with RP 4 ± 2, *p* = 0.99; TGF-α: 2.0 ± 0.5, with RP 1.2 ± 0.2, *p* = 0.33; GROα: 1.2 ± 0.2, with RP 1.1 ± 0.2, *p* > 0.99). Whereas Nguyen et al. [5] found that an increase in mRNA abundance in HAE cultures by IL-13 required co-stimulation with beta-agonist, our data show that IL-13, without beta-agonist or ALB co-treatment, was sufficient to stimulate cytokine induction > 1.5 fold (relative to Veh treatment alone) (Table 1). No difference in the regulatory effect of IL-13 or beta-agonists ALB or RP on cytokines was observed between cultures derived from asthmatic or non-asthmatic donors.

**Table 1:**
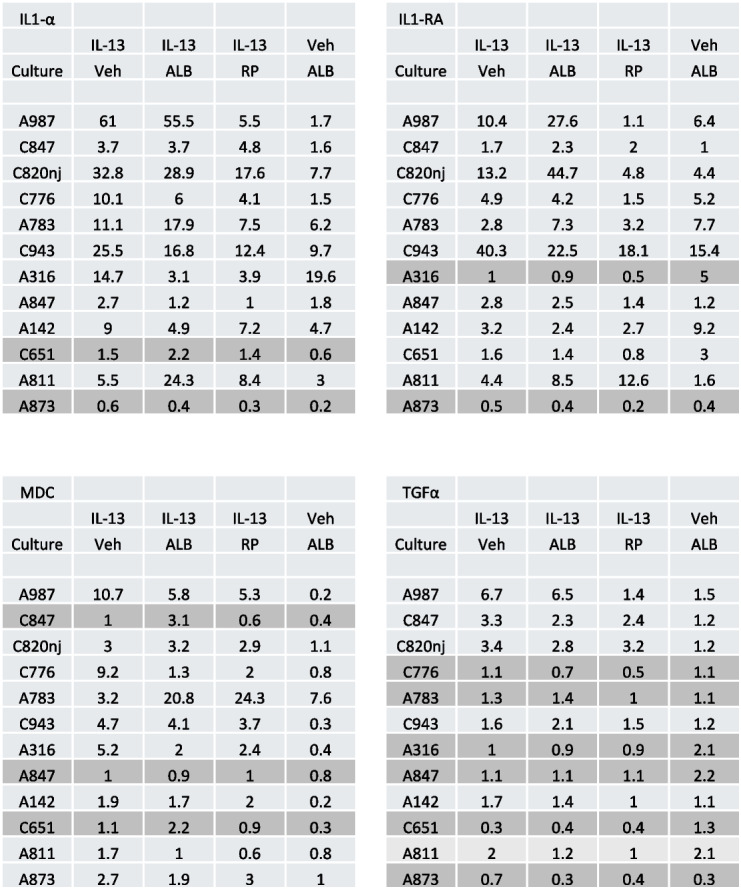
Effects of albuterol (ALB) and ractopamine (RP) on IL-13-indueced cytokines. HAE cultures were treated with IL-13 plus vehicle, 1 μM ALB or 1 μM RP. The culture media were collected and analyzed for IL-1α, MDC, TGFα, and IL-1RA expression by antibody-based multiplexing laser beads. Table shows cytokine levels normalized to basal for each of the cultures derived from 12 different human donors. For each of the cytokines assessed, cultures in which IL-13 + Veh failed to increase levels > 1.5 fold are highlighted by gray shade

## Discussion

Our findings reveal that the balanced beta-agonist ALB augments IL-13-induced mucin production, whereas the Gs-biased beta-agonist RP does not, implicating a required role of arrestin signaling by the β_2_AR. The regulatory effect of ALB on IL-13-induced cytokine induction in HAE was highly variable, having no significant effect of IL-13-induced levels on any of the cytokines examined. Further analysis of cytokine data suggests differential sensitivity among cultures to specific β_2_AR signaling arms, underscoring the considerable biologic variability among asthma patients that complicates translational asthma research.

The current study was prompted by several factors relevant to asthma safety and asthma management efficacy. A long history of asthma safety concerns exists involving beta-agonist therapy, highlighted by multiple “epidemics” associated with different therapeutic beta-agonists (reviewed in [13, 26]), and the Salmeterol Multicenter Asthma Research Trial terminated in 2003 that resulted in an FDA-mandated black box warning for drugs containing long-acting beta-agonist (LABA) [27]. Although, as noted above, subsequent studies analyzing the safety of combination therapies containing LABAs plus corticosteroids have mitigated LABA safety concerns (and contributed to the withdrawal of black box warnings). However, the efficacy of beta-agonists (both short-acting beta-agonists (SABAs) and LABAs) as asthma drugs remains a highly researched topic. As noted above, a large percentage of asthmatics have suboptimal control with the current armament of asthma drugs, and numerous studies employing murine models of allergic lung inflammation demonstrate a permissive or exacerbation effect of endogenous (epinephrine) or exogenous beta-agonist on allergen-induced lung inflammation [4–6, 9].

In all murine studies of allergic lung inflammation to date assessing the effect of either endogenous or exogenous beta-agonist, or β_2_AR antagonism or ADBR2 gene ablation, beta-agonist-stimulated β_2_AR played a critical role in the increase in lung cytokine expression caused by allergen or IL-13 (reviewed in [9, 28, 29]). Not coincidently, deaths from acute asthma attacks are most frequently the result of mucus plugging [30, 31], consistent with the current finding that beta-agonist (in this case, albuterol) significantly augments MUC5AC secretion in IL-13-stimulated HAE. These findings, as well as the finding of Nguyen et al. that in HAE cultures epinephrine was required for the induction of cytokines (CCL24, CXCL1, CCL2, CCL20) by IL-13 [5], suggested a more comprehensive effect of β_2_AR agonism on lung inflammation involving both augmentation of mucin production and an immunomodulatory effect exacerbating lung inflammation.

Unresolved has been the question regarding what target cell(s) mediate this pro-inflammatory effect of beta-agonist in the lung. It is likely that both lung structural cells and infiltrating immune cells participate in arrestin-dependent, beta-agonist-mediated pro-inflammatory signaling [10]. Within those 2 broad categories of cell types, airway epithelia (structural) and T cells (infiltrating) are the most likely cell candidates, owing to their proximal involvement, both individually and in concert, in disease pathogenesis. Asthma is a highly heterogeneous inflammatory disease of the airways characterized by several different endotypes, each of which is defined by the T cell subsets involved and the cytokines they produce [32, 33]. Allergen-activated T cells propagate lung inflammation through action of their cytokines on numerous other cells, including immune cells (B cells, mast cells, eosinophils, neutrophils) and lung structural cells (airway epithelial cells, airway fibroblasts and airway smooth muscle cells) [34]. Cytokines IL-4, IL-5, IL-13, IL-9 and IL-17A/F released from Th2, Th9 and Th17 subsets [34] stimulate AE mucin [28–33] and cytokine production [34–37]. Whether T cells are involved in the pro-inflammatory effects of beta-agonist in asthma is presently unclear. Several studies show that T cell function becomes pro-inflammatory in response to β_2_AR-mediated signaling under experimental conditions that model asthma [38–40]. Using similar experimental conditions, at least one study showed contrasting findings where beta-agonists inhibit the proliferative response of human (CD4 +, CD8 +, or memory subset) T cells to anti-CD3 mAb [41]. Additionally, in a non-asthma context, evidence suggests that β_2_AR activation promotes anti-inflammatory T cell function [42–44]. Because the T cell response to beta-agonist in asthma likely differs depending on the differentiation state of the T cell, timing of the β_2_AR activation, molecular signaling pathway activated, and cytokine microenvironment [45], further investigation is required to draw any conclusions as to the immunomodulatory effect of beta-agonism on T cells in asthma. Given that interactions among several cell types are required for the full development of the asthma phenotype, paired with the fact that each of the involved cell types express functional levels of β_2_AR, it becomes important to consider the role of cells other than epithelia or T cells in mediating the pro-inflammatory effect of inhaled beta-agonists. For an excellent review on this topic see Suh and Johnston [46].

A second important finding presented herein is the ability of balanced beta-agonist, ALB, to augment the induction of mucin, whereas the Gs-biased agonist RP could not. Given previous studies established that RP is able to promote β_2_AR/Gs signaling while unable to recruit arrestin to the β_2_AR (thus defining RP as Gs-biased), the differential effect of ALB versus RP in regulating mucin is likely attributed to ALB-stimulated arrestin signaling, or arrestin-dependent regulation of the ALB-stimulated β_2_AR. Such an interpretation is consistent with our studies demonstrating the ability of strategies either blocking arrestin effects [10, 11] or selectively augmenting cAMP/PKA signaling via phosphodiesterase inhibition [4] to mitigate allergic lung inflammation.

Whereas our findings regarding mucin regulation support the premise that airway epithelial cells contribute to the previously-demonstrated pro-inflammatory effects of β_2_AR agonism in vivo, the results with respect to regulation of cytokine secretion were decidedly equivocal. We were unable to establish any clear regulatory effect of (biased or balanced) beta-agonists on IL-13-induced cytokine production in human airway epithelia cultures, most likely due to the considerable variability of response among cultures. Interestingly, the requirement of concomitant β_2_AR agonism in the IL-13-stimulated induction of inflammatory cytokines in HAE, previously reported in Nguyen et al. [5] was only observed in a minority of cultures (Table 1) examined in the present study, suggesting the existence of distinct beta-agonist -sensitive and -insensitive populations/endotypes. Analyses of HAE in Nguyen et al. relied on a singular, commercially-obtained culture, whereas the present study took advantage of numerous HAE cultures generated from donors participating in clinical research studies at National Jewish Health. Our findings suggest that the exacerbating effect of beta-agonist on inflammation-induced cytokine production in epithelia may be limited to a subset of asthmatics, consistent with the high degree of variability in clinical research data assessing lung inflammation in asthmatic subjects [47, 48]. They further suggest (as discussed above), that for many if not all asthmatics, the direct effect of beta-agonists on airway epithelia is insufficient to create the significant augmenting effect on cytokine induction observed in vivo, this in vivo effect requiring a cooperativity of effects on multiple cell types.

Certain limitations of the current study should be noted. One, the study was not exhaustive in the analysis of cytokines whose induction by IL-13 are potentially regulated by beta-agonists, having focused on a handful of cytokines that preliminary studies identified as among the most induced by IL-13. A more expansive analysis may yield positive findings for additional cytokines. Two, we identified a positive regulatory effect of ALB alone (in the absence of IL-13) on mucin induction, yet did not explore the mechanism underlying this effect, or examine whether RP alone could similarly induce mucin production. This limitation arose given our a priori hypothesis was that ALB, but not RP, augments IL-13-induced mucin; a regulatory effect of beta-agonist alone had no conceptual or empirical basis, so an experimental design that could explore a potential mechanism, or include analysis of the effect of RP alone, was not considered. Clearly, this intriguing effect of ALB in the absence of IL-13 merits future investigation.

A final issue worth discussing is whether our findings with respect to albuterol observed in the current study can be generalized to all clinically used SABAs and LABAs. Although albuterol is a frequently-used beta-agonist, particularly for rescue from acute exacerbations, various LABAs are used in maintenance combination therapy (i.e., LABA plus inhaled corticosteroid) as recommended by current GINA guidelines [49]. Because the specific degree of “bias” differs among beta-agonists [50], the extent to which any given beta-agonist augments IL-13-induced mucin production in human airway epithelia would need to established empirically. Presumably, agonists that are balanced would augment similar to the effect observed with albuterol, whereas those skewing towards Gs bias may not.

In summary, the current study implicates airway epithelia as a critical target cell through which beta-agonists exacerbate mucin production during allergic lung inflammation. Further, based on comparison of effects of balanced versus Gs-biased beta-agonists, the arrestin-dependent signaling arm of the β_2_AR appears to mediate this exacerbating effect. Finally, the lack of effect of beta-agonists on IL-13-stimulated cytokine production in HAE cultures suggests that the comprehensive effect of beta-agonists on allergic lung inflammation likely involves a complex, integrative mechanism involving multiple cell types.

## Supplementary Information

Supplementary Material 1.

## Data Availability

The datasets used and/or analyzed during the current study are available from the corresponding author on reasonable request.

## Abbreviations

6CKine: Chemokine (C–C motif) ligand 21 (CCL21)
3T3: Mouse embryonic fibroblast (3T3) feeder cells
AE: Airway epithelium/airway epithelial cells
ALB: Albuterol
ALI: Air–liquid interface
ANOVA: Analysis of variance
BCA-1: B cell-attracting chemokine 1 (CXCL13)
BRET: Bioluminescence resonance energy transfer
cAMP: Cyclic adenosine monophosphate
CCL2: Chemokine (C–C motif) ligand 2
CCL20: Chemokine (C–C motif) ligand 20
CCL22: Chemokine (C–C motif) ligand 22 (MDC)
CCL24: Chemokine (C–C motif) ligand 24
CD4: Cluster of differentiation 4
CD8: Cluster of differentiation 8
CTCK: Cutaneous T cell-attracting chemokine (CCL27)
CXCL1: Chemokine (C–X–C motif) ligand 1 (GROα)
DMEM: Dulbecco’s modified Eagle medium
EGF: Epidermal growth factor
ENA-78: Epithelial-derived neutrophil-activating peptide 78 (CXCL5)
EP2: Prostaglandin E₂ receptor subtype 2
FGF-2: Fibroblast growth factor 2
Flt-3 ligand: Fms-like tyrosine kinase 3 ligand (Flt3L)
G-CSF: Granulocyte colony-stimulating factor
GINA: Global Initiative for Asthma
GM-CSF: Granulocyte–macrophage colony-stimulating factor
GROα: Growth-regulated oncogene alpha (CXCL1)
Gs: Stimulatory G protein alpha subunit (Gαs)
HAE: Human airway epithelia (airway epithelial cultures)
HEK293: Human embryonic kidney 293 cells
IFNα2: Interferon alpha 2
IFNγ: Interferon gamma
IL: Interleukin
IP-10: Interferon gamma-induced protein 10 (CXCL10)
IRB: Institutional review board
LABA: Long-acting beta-agonist
LIF: Leukemia inhibitory factor
M-CSF: Macrophage colony-stimulating factor
MCP: Monocyte chemoattractant protein
MDC: Macrophage-derived chemokine (CCL22)
mAb: Monoclonal antibody
MIP: Macrophage inflammatory protein
MUC5AC: Mucin 5AC
NAM: Negative allosteric modulator
NIH: National Institutes of Health
NJH: National Jewish Health
NP40: Nonidet P-40 (detergent)
OGRI/OGR1: Ovarian cancer G protein-coupled receptor 1 (GPR68; proton-sensing receptor)
PAM: Positive allosteric modulator
PAR2: Protease-activated receptor 2
PBS: Phosphate-buffered saline
PDE4: Phosphodiesterase 4
PDGF: Platelet-derived growth factor
PKA: Protein kinase A
PNMT-KO: Phenylethanolamine N-methyltransferase knockout
pVASP: Phosphorylated vasodilator-stimulated phosphoprotein
RANTES: Regulated upon activation, normal T cell expressed and secreted (CCL5)
RIPA: Radioimmunoprecipitation assay (buffer)
RP: Ractopamine
SABA: Short-acting beta-agonist
SCF: Stem cell factor
SDF-1α/β: Stromal cell-derived factor 1 alpha/beta (CXCL12)
SDS: Sodium dodecyl sulfate
SEM: Standard error of the mean
sCD40L: Soluble CD40 ligand
TARC: Thymus and activation-regulated chemokine (CCL17)
TGF-α: Transforming growth factor alpha
Th2: T helper type 2
Th9: T helper type 9
Th17: T helper type 17
Thymic stromal lymphopoietin (TSLP): Thymic stromal lymphopoietin
TNF: Tumor necrosis factor
TJU: Thomas Jefferson University
TPO: Thrombopoietin
TRAIL: TNF-related apoptosis-inducing ligand
TSLP: Thymic stromal lymphopoietin
VASP: Vasodilator-stimulated phosphoprotein
VEGF-A: Vascular endothelial growth factor A
β-actin: Beta-actin
β_2_AR: Beta-2-adrenoceptor
