# Supplementary material for: Qualitative beta-2-adrenoceptor signaling in the regulation of human airway epithelia mucin and cytokine production
Source: Respir Res. 2026 May 1;27:261. doi: 10.1186/s12931-026-03693-4 (PMC13312746; doi:10.1186/s12931-026-03693-4)
Supplement: Supplementary file 1 — Supplementary Material 1. [file 12931_2026_3693_MOESM1_ESM.docx]

**Supplemental Figure:**
